# Transcriptome-Wide Identification, Classification, and Characterization of *AP2/ERF* Family Genes in the Desert Moss *Syntrichia caninervis*

**DOI:** 10.3389/fpls.2017.00262

**Published:** 2017-02-27

**Authors:** Xiaoshuang Li, Daoyuan Zhang, Bei Gao, Yuqing Liang, Honglan Yang, Yucheng Wang, Andrew J. Wood

**Affiliations:** ^1^Key Laboratory of Biogeography and Bioresource in Arid Land, Xinjiang Institute of Ecology and Geography, Chinese Academy of SciencesUrumqi, China; ^2^State Key Laboratory of Agrobiotechnology, School of Life Sciences, The Chinese University of Hong KongHong Kong, China; ^3^University of Chinese Academy of SciencesBeijing, China; ^4^Department of Plant Biology, Southern Illinois University, CarbondaleIL, USA

**Keywords:** *AP2/ERF* genes, *Syntrichia caninervis*, transcriptome, classification, desiccation tolerance

## Abstract

APETALA2/Ethylene Responsive Factor (AP2/ERF) is a large family of plant transcription factors which play important roles in the control of plant metabolism and development as well as responses to various biotic and abiotic stresses. The desert moss *Syntrichia caninervis*, due to its robust and comprehensive stress tolerance, is a promising organism for the identification of stress-related genes. Using *S. caninervis* transcriptome data, 80 AP2/ERF unigenes were identified by HMM modeling and BLASTP searching. Based on the number of AP2 domains, multiple sequence alignment, motif analysis, and gene tree construction, *ScAP2/ERF* genes were classified into three main subfamilies (including 5 *AP2* gene members, 72 *ERF* gene members, and 1 *RAV* member) and two Soloist members. We found that the ratio for each subfamily was constant between *S. caninervis* and the model moss *Physcomitrella patens*, however, as compared to the angiosperm Arabidopsis, the percentage of ERF subfamily members in both moss species were greatly expanded, while the members of the AP2 and RAV subfamilies were reduced accordingly. The amino acid composition of the AP2 domain of ScAP2/ERFs was conserved as compared with Arabidopsis. Interestingly, most of the identified *DREB* genes in *S. caninervis* belonged to the A-5 group which play important roles in stress responses and are rarely reported in the literature. Expression profile analysis of *ScDREB* genes showed different gene expression patterns under dehydration and rehydration; the majority of *ScDREB* genes demonstrated a stronger response to dehydration relative to rehydration indicating that *ScDREB* may play an important role in dehydrated moss tissues. To our knowledge, this is the first study to detail the identification and characterization of the AP2/ERF gene family in a desert moss. Further, this study will lay the foundation for further functional analysis of these genes, provide greater insight to the stress tolerance mechanisms in *S. caninervis* and provide a reference for AP2/ERF gene family classification in other moss species.

## Introduction

*Syntrichia caninervis* is a dominant moss species of biological soil crusts in the Gurbantunggut desert of Northwestern China (Zhang, 2005). *S. caninervis* has gained increasing attention due to a comprehensive tolerance to stresses such as desiccation, elevated temperature, low temperature, and high radiation. Studies on *S. caninervis* have been focused on the morphology (Stark et al., 2004, 2005; Xu et al., 2009a; Zheng et al., 2011; Tao and Zhang, 2012; Pan et al., 2016), physiology (Xu et al., 2009b; Li et al., 2010; Zhang et al., 2011; Wu et al., 2012; Yin and Zhang, 2016), and response to DT. *S. caninervis* is being developed as a model moss for studying the molecular mechanisms of DT and a good plant material for identification of stress-related genes (Wood, 2007; Yang et al., 2012, 2015; Li et al., 2015).

APETALA2/Ethylene Responsive Factor (AP2/ERF) is a large family of plant TFs, containing at least one DNA-binding domain (AP2 domain), which play important roles in the control of primary metabolism, secondary metabolism, and development as well as response to various biotic and abiotic stresses (Licausi et al., 2013). Two main classification methods have been proposed for the plant AP2/ERF superfamily based upon sequence similarities and the number of AP2 domains. Sakuma et al. (2002) classified the AP2/ERF superfamily into five families: AP2, RAV, DREB, ERF, and Soloists. The ERF family is also known as the EREBP (ethylene-responsive element binding proteins) family (Nakano et al., 2006). The AP2 family contains two AP2 domains, the RAV family contains one AP2 domain and one B3 domain, while the DREB, ERF, and Soloists families each contain a single AP2 domain. Furthermore, according to sequence similarity of the single AP2 domain, *DREB* family genes are further classified into the groups A1 to A6 and *ERF* family genes are divided into the groups B1 to B6 (Sakuma et al., 2002). Nakano et al. (2006) classified AP2/ERF predicted amino acid sequences proteins into three major families: AP2, ERF (include both DREBs and ERFs), and RAV. The ERF family was sub-divided into 12 groups in Arabidopsis and fifteen groups in rice according to the structure and similarity of the AP2 domain (Nakano et al., 2006). Both classification schemes have been extensively employed in the literature, and the methods have direct correspondence. For example, the A-1 type of DREB family in Sakuma‘s classification corresponds to the Group III ERF family in Nakano’s classification (Nakano et al., 2006).

*APETALA2/Ethylene Responsive Factor* superfamily genes and transcripts have been identified and characterized in many plants and the superfamily has been studied extensively in the context of plant stress tolerance (Xu et al., 2011; Mizoi et al., 2012). *DREB* family genes have been documented to respond to drought, desiccation, osmotic stress, salt, low temperature (cold) and elevated temperature (heat) and are candidate genes for improving plant stress tolerance in crop plants (Lata and Prasad, 2011). The genome-wide identification of the AP2/ERF gene family has been accomplished in a number of plant species including Arabidopsis (Czechowski et al., 2005), rice (Narsai et al., 2010), poplar (Zhuang et al., 2008), grape (Licausi et al., 2010), soybean (Zhang et al., 2008), castor bean (Xu et al., 2013), cotton (Lei et al., 2016), lotus (Sun et al., 2014), Chinese cabbage (Song et al., 2013), *Medicago truncatula* (Shu et al., 2015), and Musa species (Lakhwani et al., 2016). Using transcriptome and EST data, *AP2/ERF* superfamily genes has been analyzed in *Brassica* ssp. (Zhuang et al., 2010; Zhuang and Zhu, 2014), *Triticum aestivum* (Zhuang et al., 2011), *Hevea brasiliensis* (Duan et al., 2013), and tea (Wu et al., 2015). Gao et al. (2014) generated the first large scale transcriptome dataset for *S. caninervis* consisting of 92,240 unigenes. In this study, we identified 80 *AP2/ERF* genes within the *S. caninervis* transcriptome. Based upon phylogenetic and protein motif structural analyses, 80 members of the ScAP2/ERF family were classified using the Arabidopsis AP2/ERF superfamily classification as reference. The expression pattern of *ScERF* genes were analyzed using RT-qPCR in response to a DT-plant specific dehydration-rehydration protocol employed by our research group (Li et al., 2015). This study is the first identification, classification, and characterization of the AP2/ERF gene family in a moss species. As such, it will lay the foundation for further functional analysis of these *AP2/ERF* family genes and provide valuable information in understanding the molecular mechanisms of the stress response in *S. caninervis*.

## Materials and Methods

### Annotation of the AP2/ERF Protein Families in *S. caninervis*

Previously, we established a comprehensive transcriptome of *S. caninervis* transcripts during a standardized dehydration-rehydration process (Gao et al., 2014). Ninety-two thousand, two hundred and forty *S. caninervis* unigenes were obtained and served as the source for the *AP2/ERF* genes identification presented in this study. Arabidopsis and *Physcomitrella patens AP2/ERF* family genes were downloaded from the plant TF database (PlantTFDB v3.0)^1^ (Jin et al., 2014). The HMM profiles were downloaded from Pfam database 27.0^2^ (Punta et al., 2012).

One hundred and seventy-six Arabidopsis AP2 predicted amino acid sequences and one hundred and seventy-one *P. patens* AP2 predicted amino acid sequences were used as queries to search against the *S. caninervis* transcriptome database using BLASTP programmer, and an *E* value of 1e-3 was adopted. Next, the HMM profile PF00847 (AP2 domain) and PF02362 (B3 domain) were queried using hmm search command included in the HMMER (v3.0) software with an *E* value cutoff at 1e-3. All the candidate *ScAP2/ERF* genes identified through these two methods were confirmed with Conserved Domain Database (CDD^3^; Marchler-Bauer et al., 2015) and SMART^4^ (Letunic et al., 2015) search to ensure the presence of AP2 domain. An AP2 domain length of approximately 60 amino acids was considered to be a full-length AP2 domain (Duan et al., 2013). Only sequences longer than 200 nucleotides were retained for further analysis. Sequences which shared >95% matches were considered redundant. The detailed flowchart of *AP2/ERF* gene detection in *S. caninervis* is depicted in **Supplementary Figure S1**.

### Sequence Analysis and Phylogenetic Tree Construction

Open reading frames (ORFs) were predicted with the ORF Finder at NCBI^5^. Motif detection was performed with the online tool MEME program^6^ (Bailey et al., 2009) using the parameters: any number of repetitions per sequence, motif width ranges of 6–50 amino acids, and 11 as the maximum number of motif. The motif alignment was conducted using MAST^7^ and Patmatch programs^8^ (Bailey et al., 2009). Physical and chemical characterization of genes with deduced amino acid sequence were analyzed using ProtParam^9^ and protein subcellular location(s) were predicted using PSORT^10^. Multiple sequence alignment was performed with ClustalW in conjunction with MEGA 5.1 (Tamura et al., 2011), phylogenetic trees were constructed by the neighbor-joining (NJ) method using MEGA 5.1 (Poisson correction and pairwise deletion). Support for nodes on the estimated phylogeny was tested with 1000 bootstrap replicates.

### Gene Expression Analysis in Dehydration-Rehydration Process

#### Primers for Real-Time PCR

Reverse transcription quantitative real-time polymerase chain reaction primers were designed with Primer Premier 5.0. To verify primer specificity, the designed primer sets were searched using BLAST against the local transcriptional data of *S. caninervis*. The primer sets were further assessed using melting-curve analysis after RT-qPCR and gel electrophoresis analysis of the amplicons.

#### Plant Material Treatment

*Syntrichia caninervis* gametophytes were collected from the Gurbantunggut Desert of Xinjiang Uyghur Autonomous Region of China (Fukang County, 44°32′30″N, 88°6′42″E) as described by Li et al. (2015). For the desiccation-rehydration process, dry gametophores were fully hydrated with MINIQ-filtered water for 24 h (which served as the control), followed by desiccation at room temperature in glass desiccators (using activated silica gel as desiccant) at 25°C (Yang et al., 2012). Samples were collected after 0.5, 2, 6, 12, and 24 h of dehydration. Samples were subsequently rehydrated by transferring the plants to new Petri plates and the filter paper was saturated with 8 mL filtered water at 25°C; hydrated samples were harvested after 0.5, 2, 6, 12, and 24 h rehydration.

#### RNA Extract and cDNA Synthesis

Total RNA was extracted using RNAiso reagent (Takara, Japan). Genomic DNA contamination was eliminated using RNase-free DNaseI (Takara, Japan). RNA quality was determined using gel electrophoresis and a NanoDrop ND-2000 spectrophotometer (Thermo Fisher Scientific, USA). The 260/280 ratio of RNA samples between 1.8 and 2.1, and 260/230 ratio higher than 1.8 were used for subsequent experiment. First strand cDNA was synthesized using PrimeScript^TM^ RT reagent kit (Takara, Japan) according to the instruction. cDNAs qualities were tested as templates by amplifying the *ScACT* gene using RT-PCR (data not shown). All cDNA was stored at -20°C prior to use.

#### RT-qPCR Assay and Data Analysis

RT-qPCR reactions were carried out using CFX96 Real-Time PCR Detection System (Bio-Rad, USA) and SYBR *Premix Ex Taq^TM^* kit (Takara, Japan). The reaction mixture consisted of 2 μl 1:5 diluted cDNA samples, 0.4 μl each of the forward and reverse primers (10 μM), 10 μl real-time master mix, and 7.2 μl water in a final volume of 20 μl. Three biological replicates and three technical replicates of each biological replicate with a no-template control (NTC) were also used. The RT-qPCR protocol was as follows: 30 s initial denaturation at 95°C, 40 cycles of 94°C for 5 s, and 58–60°C for 30 s. The relative expression levels of genes were calculated relative to the control (fully hydrated samples). The combination of *ScACT* and *Sc*α*-TUB* are used to reliably normalize the RT-qPCR data based upon our previous research (Li et al., 2015).

### Statistical Analysis

Statistical analyses were performed using SPSS software (standard version 11.5 released for Windows, SPSS Inc., Chicago, IL, USA). All data were analyzed using a one-way analysis of variance (ANOVA) at the 95% confidence level. The significant difference was compared using LSD multiple comparison test. The data shown are the mean values ± SE of three replicates, and the significance level relative to controls is ^∗^*P* < 0.05, ^∗∗^*P* < 0.01. Figures were generated using Sigmaplot10.0. Adobe Illustrator CS5 and Adobe Photoshop CS3 were used for image processing.

## Results

### Identification and Classification of AP2/ERF Genes

Eighty AP2/ERF unigenes were identified from the *S. caninervis* transcriptome using HMMs and BLASTP (see **Supplementary Figure S1** for the detailed screening strategy). The unigenes ranged from 202 to 2514 bp in length, and the corresponding predicted amino acid sequences ranged from 39 to 659 aa. AP2 domain prediction of the *ScAP2/ERF* genes demonstrated that 68 of the 80 unigenes (85%) contained a full-length AP2 domain (ca 60 aa), five unigenes contained two AP2 domains, and a single unigene contained both a single AP2 domain and a B3 domain. Seventeen of the 80 unigenes (21%) contained an intact ORF. Based on these results, the 74 unigenes encoding a single AP2 domain which were preliminarily classified as members of the ERF protein family (some special AP2 family protein members and Soloist proteins also contain a single AP2 domain). The five AP2 double-domain unigenes were classified as AP2 protein family members and the AP2/B3 unigene was classified as a RAV protein family member. Group classification of the *ScAP2/ERF* genes was accomplished by constructing a phylogenetic gene tree which aligned the predicted AP2 domains using the NJ method. Nine unigenes were excluded from further analysis due to truncated AP2 domains, finally 71 predicted amino acid sequences were used to construct the phylogenetic tree. The un-rooted gene tree demonstrated that the 71 *S. caninervis* AP2/ERF predicted amino acid sequences can be divided into five main groups: ERF I, ERF II, ERF III, AP2, and RAV (**Supplementary Figure S2**).

**FIGURE 1:**
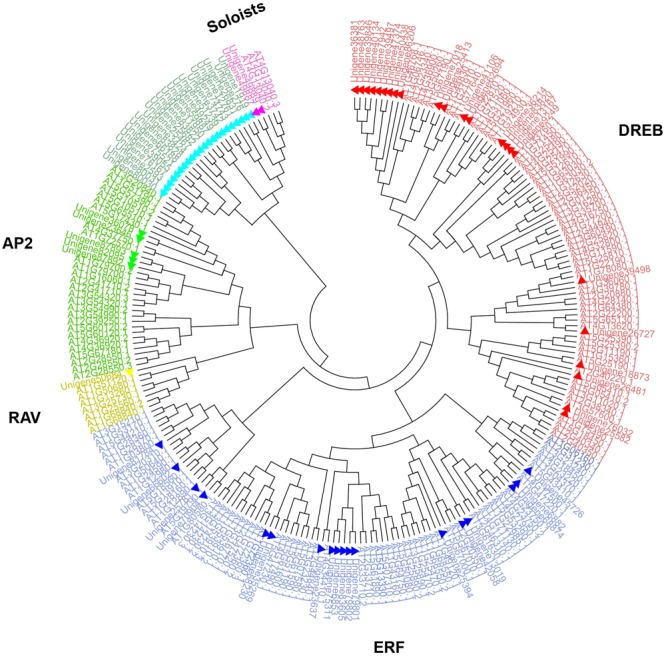
**Phylogenetic analysis of *AP2/ERF* family genes in *Syntrichia caninervis* and Arabidopsis.** The gene tree was constructed using the neighbor-joining (NJ) method using 71 ScAP2/ERFs and 176 AtAP2/ERFs, Poisson model with pairwise deletion. Bootstrap values from 1000 replicates were indicated on the side of the node and used to assess the robustness of the tree. To distinguish ScAP2/ERFs from AtAP2/ERFs, ScAP2/ERF were marked with triangles. Different subfamily genes were marked and grouped in different colors (AP2, ERF, DREB, RAV, and Soloists subfamilies were labeled in green, blue, red, yellow, and purple, respectively).

Sixty-five unigenes containing a single AP2 domain grouped into three clades: ERF I (two sequences), ERF II (41 sequences), and ERF III (22 sequences). Five unigenes which contained two AP2 domains grouped into the same clade (AP2), and the AP2/B3 unigene formed a unique clade (RAV). Additional phylogenetic analysis employing 71 *ScAP2/ERF* predicted amino acid sequences and 176 previously annotated Arabidopsis *AP2/ERF* predicted amino acid sequences supported the classification into five groups (**Figure 1**). The number of *ScAP2/ERF* subfamily genes were compared with the model plants Arabidopsis and *P. patens*. The ERF subfamily was the largest in each species ranging from 77 to 90% (**Table 1**), while RAV and Soloist subfamily was the smallest ranging from 1 to 4%.

**Table 1:** Distribution of *APETALA2 (AP2)/ERF* genes in Arabidopsis, *Physcomitrella patens*, and *Syntrichia caninervis.*

Subfamily	*A. thaliana* genome		*P. patens* genome		*S. caninervis* transcriptome	
	Number	Ratio	Number	Ratio	Number	Ratio
AP2 subfamily	30	17%	13	8%	5	6%
ERF subfamily	136	77%	154	90%	72	90%
RAV subfamily	7	4%	2	1%	1	1%
Soloist	3	2%	2	1%	2	3%
Total	176		171		80	

*Gene numbers of each subfamily conform to the PlantTFDB database (V3.0).*

### Classification of *ScERF* Genes

**FIGURE 2:**
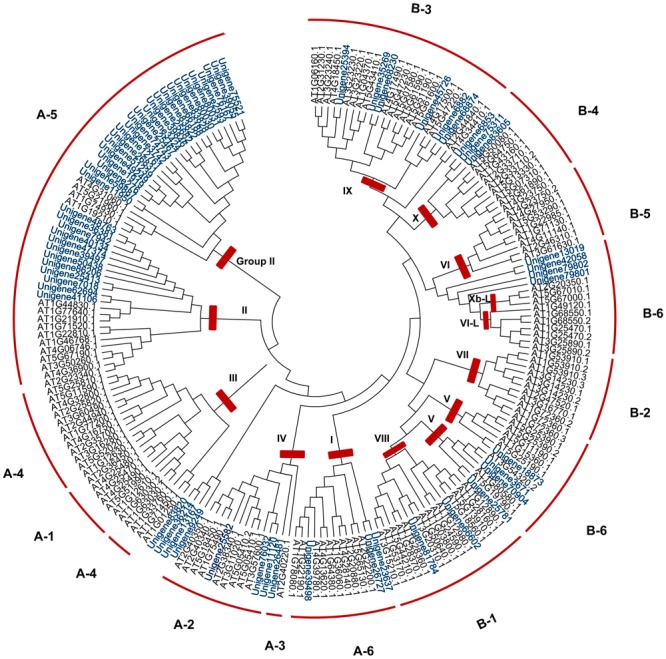
**Phylogenetic analysis of *ERF* family genes in *S. caninervis* and Arabidopsis.** The gene tree was constructed using the NJ method using 63 ScERFs and 136 AtERFs, Poisson model with pairwise deletion. To assess the robustness of the tree, bootstrap values from 1000 replicates were indicated on the side of the node. To distinguish ScERFs from AtERFs, ScERF were marked in blue. Previously reported subfamily names (A1–6, B1–6) and group names (group I-Xb-L) were employed (Sakuma et al., 2002; Nakano et al., 2006).

Within the plant AP2/ERF family, the ERF subfamily is dominant and has been extensively documented to play important roles in plant responses to biotic and abiotic stresses (Zhuang et al., 2008; Huang et al., 2016). Phylogenetic analysis using 63 ScERFs and 136 AtERFs (both of which excluded soloists) identified members of eight out of 12 subfamilies based upon the classification of Sakuma et al. (2002): A2, A3, A4, A6, B1, B3, B4, and B6. ScERF predicted amino acid sequences contained no members of the A1, A4, B2, and B5 subfamilies (**Figure 2** and **Supplementary Figure S3**). Accordingly, based upon Nakano et al. (2006)’s classification method, *ScERF* genes contained members of group I to group Xb-L, with the exception of group II, III and group VI. Most plants have a single A-3 type DREB (Sakuma et al., 2002; Zhang et al., 2008) while some species (such as grape) lack A-3 type of DREBs (Licausi et al., 2010). Unigene26481 clustered together with both A-2 and A-3 type of *AtDREBs* (**Figure 2**) which prevented a definitive classification. Additional analysis using 57 *AtDREBs* generated a tree that showed unigene26481 was clearly grouped together with the A-3 type DREB in Arabidopsis (**Supplementary Figure S3**). *S. caninervis* has a single A-3 DREB protein. Interestingly, more than half of the ERF sequences are members of the A-5 subgroup *DREBs* in *S. caninervis* while other subgroups contain between two and six sequences (**Figure 2** and **Supplementary Figure S3**).

### Conserved Amino Acid Residues in the AP2 Domain of *ScERF* Subfamily

**FIGURE 3:**
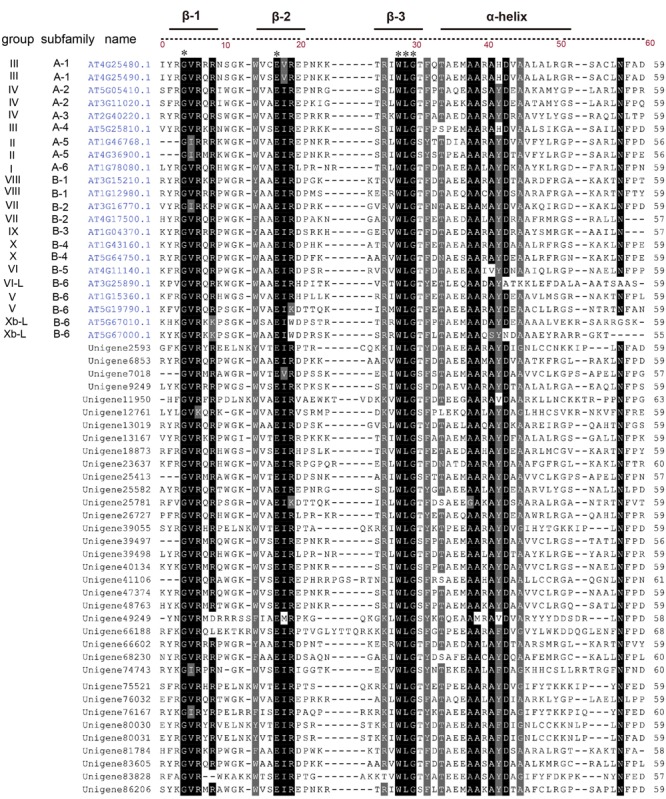
**Sequence alignments of AP2 domains of representative ERF proteins in *S. caninervis* and Arabidopsis.** Twenty-two *AtERF* genes representative of each ERF subfamily/group were aligned with *ScERFs*. The subfamily and corresponding group for each representative AtERFs is depicted on the left. The locus names of AtERFs were marked in blue, and the unigene numbers of ScERFs were labeled with a dark color. The black and light gray shading indicate identical and conserved amino acid residues. The complete conserved amino acids residues of AtERFs and ScERFs were labeled with an asterisk. The three β-sheets regions and α-helix region were labeled.

**FIGURE 4:**
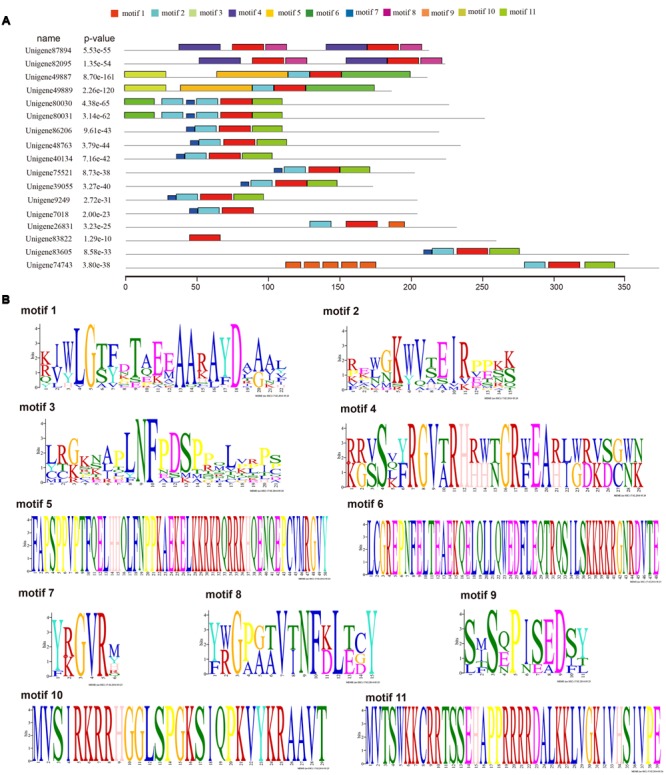
**Motif analyses of ScAP2/ERF with intact Open reading frames (ORFs) using MEME online software.** Seventeen ScAP2/ERF proteins with complete ORFs were used for motif prediction. Parameters are as follows: any number of repetitions per sequence, motif width ranges of 6–50 amino acids, and 11 as the maximum number of motif. Each of the sequence has an *E*-value less than 10. **(A)** Motif composition of ScAP2/ERF proteins, **(B)** deduced amino acid sequence of each motif.

To analyze the conservation of the AP2 domains, *ScERF* deduced amino acid sequences were aligned with 22 *AtERF* sequences representing each ERF subfamily/group. Sequence alignment demonstrated that *ScERF* sequences shared significant amino acid similarity with *AtERF*s and that the AP2 domains of *ScERFs* also contained three β-sheets and one α-helix (**Figure 3**). The amino acid residues 4 G, 16 E, 27 W, 28 L, 29 G were completely conserved in all *ScERF* and *AtERF* genes (labeled with asterisk). β-sheet 1 (β-1) contains the conserved GVR element (4G, 5V, 6R) with several sequences substituting 5 I for 5V. β-sheet 2 (β-2) contains the conserved EIR element (16 E, 17 I, and 18 R). We found that in both Arabidopsis and *S. caninervis*, the amino acid residues EVR were observed only in A-1 group DREB genes, and the residues ERK were observed only in the B-6 protein subfamily (**Figure 4**). These conserved amino acid patterns may be helpful for the classification of *ERF* genes in other species. In addition, the unigene49249 in *S. caninervis* had a particular “EMR” element in this position which was not found in Arabidopsis *ERF* genes. β-sheet 3 (β-3) contained the conserved WLG element (27 W, 28 L, 29 G) which was highly conserved between Arabidopsis and *S. caninervis*, and the α-helix contained a consensus sequence AAxAxD which was conserved between Arabidopsis and *S. caninervis* (**Figure 3** and **Supplementary Figure S4**).

### Physicochemical Property, Conserved Motif, and Phylogenetic Analyses of ScAP2/ERF Proteins with Intact ORF

**FIGURE 5:**
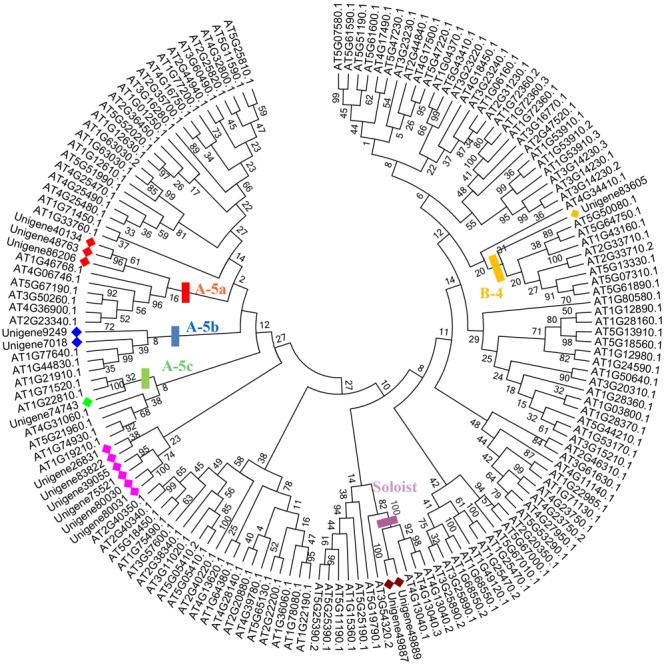
**Phylogenetic analyses of *15 ScERF* genes with intact ORFs.** The gene tree was constructed using the NJ method using 15 ScERFs with full-length ORFs and 139 AtERFs (including Soloist genes), Poisson model with pairwise deletion. Bootstrap values from 1000 replicates were indicated on the side of the node. ScERF were marked with diamonds using different colors to distinguish from AtERFs. Different clades represented specific groups were labeled with rectangles using the corresponding colors. A-5a, b, c group proteins were labeled with red, blue, and green, B-4 and Soloist groups were labeled with yellow and purple. Eight *ScERF* genes which cannot further classified were marked in pink.

Seventeen out of 80 *ScAP2/ERF* genes (2 *ScAP2*, 2 *ScSoloists*, and 13 *ScERFs*) were predicted to encode a single, intact ORF. The predicted polypeptide was characterized and the putative subcellular localization was analyzed (**Table 2**). The 17 *ScAP2/ERF* predicted polypeptides ranged from 172-to-371aa residues. The predicated molecular mass of the deduced polypeptides ranged from 19.13 to 42.27 KDa, and the theoretical pI values ranged from 4.62 to 10.0. More than half of ScAP2/ERF deduced polypeptides were predicted to localize in the nucleus (including two ScAP2). Four deduced polypeptides localized to the cytoplasm and one each localized to the microbody and plasma membrane. The 17 *ScAP2/ERF* predicted ORFs were submitted to MEME analysis to investigate the motif composition of the deduced polypeptides. In total, 11 motifs were found and their amino acids sequences were showed in **Figure 4B**. Five out of eleven motifs (motif 1, 2, 3, 7, 8) represented the AP2 domain. Motif 2 and 7 correspond to the AP2 domain β2-and β1-sheet of AP2 domain, respectively, and Motif 1 contained the β3-sheet and α-helix. Motif 3 was similar to Motif 8 which corresponded to the C-terminal of the AP2 domain (**Figure 4**). *ScERF* genes with intact ORF should be the priority candidates for gene cloning and function analysis. Therefore, we constructed the gene tree using 15 *ScERF* genes (including two Soloists) with intact ORFs and 139 Arabidopsis *ERF* genes to establish a detailed classification. The tree revealed that 15 *ScERFs* covered two ScSoloists, 12 A-5 type of DREBs, and one B-4 type of ERF gene (**Figure 5**). Three genes (unigene 40134, 48763, and 86206) belonged to A-5a subgroup, two genes (unigene 9249 and 7018) belonged to A-5b subgroup, and unigene 74743 which had the longest ORF belonged to A-5c subgroup. Additionally, six other A-5 type *ScERF* genes cannot be classified into a subgroup a, b nor c, which separately grouped in a *S. caninervis*-specific clade. One gene (unigene83605) was classified into the B-4 group.

**Table 2:** *ScAP2/ERF* deduced amino acid sequence characteristics and predicted subcellular location of genes.

Unigene number	ORF length (aa)	Molecular weight (kDa)	Theoretical pI	Location
82095	223	25.50	9.58	Nucleus (0.880)
87894	212	24.22	9.74	Nucleus (0.76)
49887	186	21.60	10.07	Nucleus (0.932)
49889	211	24.38	10.01	Plasma membrane (0.79)
7018	203	22.23	5.17	Microbody (0.558)
9249	203	22.30	5.97	Nucleus (0.98)
26831	230	25.98	8.58	Nucleus (0.76)
39055	172	19.13	9.16	Nucleus (0.3)
40134	224	24.12	5.58	Cytoplasm (0.45)
48763	234	25.04	5.8	Cytoplasm (0.45)
75311	266	28.77	5.12	Mitochondrial (0.467)
75521	201	22.46	9.58	Cytoplasm (0.45)
74743	371	42.27	5.58	Nucleus (0.70)
80030	251	27.78	5.93	Nucleus (0.88)
80031	226	25.18	8.6	Nucleus (0.867)
83605	350	37.82	5.30	Nucleus (0.30)
83822	258	29.41	4.62	Cytoplasm (0.45)
86206	219	24.04	6.45	Nucleus (0.94)

*APETALA2 genes were underlined, other genes were *ERFs* and Soloists.*

### Expression Profiles of *ScDREB* Genes Response to Dehydration-Rehydration Process

**FIGURE 6:**
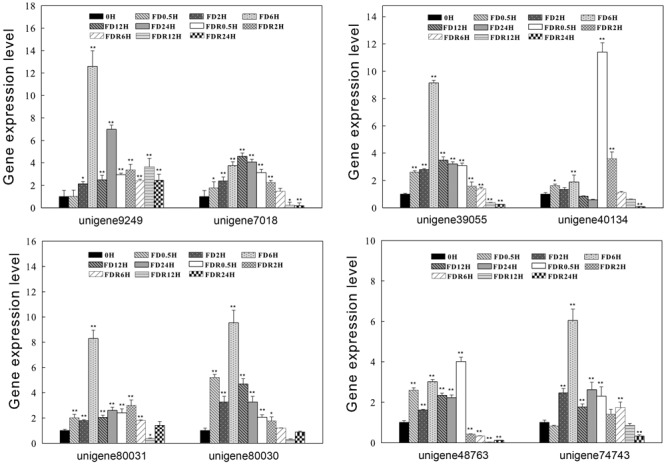
**Expression profiles of eight *ScDREB* genes during *S. caninervis* dehydration-rehydration process.** The relative gene expression levels were calculated relative to 0 h and using 2^-ΔΔCT^ method. The data shown are the mean values ± SE of three replicates, and the significance level relative to controls is ^∗^*P* < 0.05, ^∗∗^*P* < 0.01.

Based on the phylogenetic tree, eight *ScDREBs* representative of different group (such as A-5a, A-5b, A-5c) were selected to quantify the changes in transcript abundance during the dehydration-rehydration process using RT-qPCR analysis. RT-qPCR results showed that compared to full-hydration (labeled as 0 h), all eight transcripts accumulated both during the fast dry (FD) and rehydration (FDR) process (**Figure 6**). Under FD treatment, transcript abundance peaked at 6 h and then decreased, with the exception of unigene 7018 which peaked at 12 h (**Figure 6**). During FDR process, transcript abundance increased at 0.5 h and subsequently decreased, with the exception of unigenes 80030 and 9249. The majority of the tested genes have higher transcript abundance during FD compared with FDR. For example the expression level of unigene 9249 increased 12-fold at FD 6 h, but was less than 4-fold increased during rehydration, suggesting that these genes may play a dominant role in fast drying process. While for unigene 40134, which has higher expression level during rehydration process, the gene expression level increased up to 12-fold at FDR 0.5 h compared to the control, suggesting unigene 40134 may play important roles at the early stage of rehydration.

## Discussion

The *AP2/ERF* genes has been identified in many plant species due to its important roles in development, metabolism, and in response to various stresses (Mizoi et al., 2012; Licausi et al., 2013). *AP2/ERF* classification employs a well-established nomenclature established in Arabidopsis and rice (Nakano et al., 2006) and this nomenclature has been used to classify the *AP2/ERF* family genes in other plant species (Mizoi et al., 2012; Song et al., 2013). Advancements in sequencing technology and the availability of large datasets has aided the identification of *AP2/ERF* family genes in several plant species using both genomic, transcriptomic or EST data (Licausi et al., 2010; Duan et al., 2013; Zhuang and Zhu, 2014; Wu et al., 2015; Lei et al., 2016). In the model moss *P. patens AP2/ERFs* were demonstrated to be regulated in response to salinity, UV and other stresses indicating that AP2/ERF transcriptional factors also play a central regulatory role during stress response in moss species (Hiss et al., 2014).

*Syntrichia ruralis* exhibits comprehensive stress tolerance (Oliver et al., 1993; Zhang et al., 2009), and has been a good resource for identifying of genes associated with stress tolerance (Yang et al., 2012; Gao et al., 2014; Li et al., 2015; Yang et al., 2015). The AP2/ERF gene family has not been characterized in *P. patens* and previous data demonstrated that the *AP2/ERF* genes were the most abundant transcriptional factors in *S. caninervis* (Gao et al., 2014). To our knowledge the transcriptome-based identification and classification of AP2/ERF family presented here is the report on AP2/ERF family analysis in any moss species. As such it will lay the foundation for further functional analysis of these AP2/ERF genes in *S. caninervi*s and provide the reference for classification of AP2/ERF gene family in other moss species.

### ScAP2/ERFs Showed Commonalities Characteristics

Phylogenetic tree analysis showed the suitability of using the classic AtAP2/ERF-based classification method with moss derived sequence data and provides evidence that the phylogenetic topology of the AP2/ERF family had already been established before the divergence of vascular plants (Mizoi et al., 2012).

The AP2 domain of *AP2/ERF* genes was reported to be highly conserved among different plant species, although the sequence similarity outside the AP2 domain was very low (Kizis et al., 2001). Similar to other plants, our results showed that the amino acid composition of the AP2 domain was very conservative between Arabidopsis and *S. caninervis*, and the 4 G, 16 E, 27 W, 28 L, 29 G amino acids were completely conserved in all ScERFs and AtERFs. Other amino acid variation can further refine the classification of genes. For example, the motifs “HLG” and “WLG” in the β3-sheet can distinguish a *Soloist* gene from an *ERF* gene. Interestingly, we found that in β2-sheet of AP2 domain of both *S. caninervis* and Arabidopsis, the vast majority of genes shared an “EIR” element, while “EVR” existed only in the A-1 group of *DREB* genes and “ERK” was specific to the B-6 subfamily genes in this position.

### ScAP2/ERFs Showed Unique Characteristics Compared with Arabidopsis

*ScAP2/ERFs* showed commonalities as well as unique characteristics as compared with Arabidopsis. Most of *ScERF* genes can be classified as described above. A small number of *ScERF* genes form a unique clade (A-5 DREB) but cannot be classified into a subgroup. The AP2/ERF superfamily in moss encompassed all of the Arabidopsis, and the ratio for each subfamily was constant between *S. caninervis* and *P. patens*. However, compared with Arabidopsis, the percentage of ERF subfamily members in the moss species were greatly expanded, and the gene members in the AP2 and RAV subfamilies were reduced. This adjustment of subfamily members may connected with their proposed functions in both development and stress response. The AP2 subfamily is associated with plant development and ERFs play important roles in abiotic stress tolerance regulation. Abundant ERF genes in moss might play important roles in abiotic stress tolerance, which is an adaptive evolutionary adaption for plant species growing in adverse environments (Licausi et al., 2013). Genes from different group may have specific motifs. Conserved motifs outside the AP2 domain region may function as a repression or activation domain, such as ERF-associated amphiphilic repression (EAR: DLNxxP) motif (Ohta et al., 2001; Kagale and Rozwadowski, 2011), LWSY motif (Dubouzet et al., 2003; Chen et al., 2008), and DELL motif (Tiwari et al., 2012). In *ScERFs*, unigene 74743 had the longest ORF and the AP2 domain was located in the end of C-terminus and contained motif 9 (SXSZPISEDSY) which was repeated five times before the AP2 domain (**Figure 4**). Bioinformatic analysis has failed to identify ‘motif 9’ in any additional plant protein. Motif 9 is a novel sequence and the function of this motif is unclear. A priority of our future work is the functional analysis of this novel sequence.

### Studies on A-5 Type of DREB Proteins Are Rare Which Play Important Roles in Stress Response

*Dehydration-responsive element-binding proteins* genes can be divided into the A1–A6 subgroups according to the sequence similarity of AP2 domain. In Arabidopsis, A-1 (DREB1) and A-2 (DREB2) type DREB proteins have been intensively studied and were reported to play important roles in plant response to cold and osmotic stresses (Sakuma et al., 2002). DREB A-1 and DREB A-2 homologous have been identified and functional analyzed in a variety of plants (Sakuma et al., 2006; Qin et al., 2007; Cong et al., 2008; Yang et al., 2011; Mizoi et al., 2013). Despite representing a large proportion of the DREB family, A-5 type of DREB proteins are poorly studied, and the function and stress response mechanisms of A-5 subgroup proteins is still unclear. In Arabidopsis, stress-associated DREB A-5 proteins were the largest subgroup (with 16 gene members; Sakuma et al., 2002), only the *RAP2.1* gene was studied in detail (Dong and Liu, 2010). Additional A-5 DREBs were reported in cotton (Huang and Liu, 2006), soybean (Chen et al., 2007, 2009), potato (Bouaziz et al., 2012), *Malus sieversii* (Zhao et al., 2012), and *Halimodendron halodendron* (Ma et al., 2015). Based upon the literature, A-5 DREB proteins showed a diversity in gene expression (i.e., stress response) and putative function (i.e., stress tolerance). The A-5 type of *DREB*s response to at least one kind of abiotic stress, such as soybean *GmDREB3* responds to a single abiotic stress (i.e., low temperature), while *GmDREB2* and *PpDBF1* in *P. patens* can respond to drought, salt, cold, and ABA treatments (Chen et al., 2007, 2009; Liu et al., 2007). Moreover, manyA-5 type of DREB proteins have been shown to enhance stress tolerance such as GmDREB2, GmDREB3, and MsDREBA5 (Chen et al., 2007, 2009; Zhao et al., 2012). Interestingly, RAP2.1 in Arabidopsis was reported to negatively regulate drought and cold tolerance in transgenic Arabidopsis (Dong and Liu, 2010). *P. patens* has a single A-5 type *DREB* (*PpDBF1*) isolated using FDD technology (Liu et al., 2007). In this study, the vast majority of *DREB* genes found in *S. caninervis* transcriptome were A-5 type *DREBs*, and the majority respond to dehydration and/or rehydration treatment. Unlike Arabidopsis and other plants, we postulate that A-5 type DREB proteins in moss species are the dominant regulator for stress response rather than A-1 (DREB1) and A-2(DREB2) proteins. Hence, to better understand the functional mechanism of plant response to stresses, more members of A-5 subgroup DREB proteins needed to be identified and studied.

## Author Contributions

DZ planned and designed the study. BG performed the bioinformatic analysis, YL, HY, and XL executed the experiments, generated the tables and figures. XL wrote the manuscript, AW and YW revised the manuscript and especially contributed to the discussion part of the manuscript. All of the authors read and approved the final manuscript.

## Conflict of Interest Statement

The authors declare that the research was conducted in the absence of any commercial or financial relationships that could be construed as a potential conflict of interest.

## Footnotes

1 http://planttfdb.cbi.pku.edu.cn/index.php

2 http://pfam.sanger.ac.uk/

3 http://www.ncbi.nlm.nih.gov/cdd/

4 http://smart.embl-heidelberg.de/

5 http://www.ncbi.nlm.nih.gov/gorf/gorf.html

6 http://meme-suite.org/index.html

7 http://meme-suite.org//tools/mast

8 https://www.arabidopsis.org/cgi-bin/patmatch/nph-patmatch.pl

9 http://web.expasy.org/protparam/

10 http://psort.hgc.jp/form.html

## Supplementary Material

FIGURE S1 **Flowchart of identification of AP2/ERF family genes in *Syntrichia caninervis***.

FIGURE S2 **Phylogenetic analysis of *AP2/ERF* family genes in *S. caninervis*.** The gene tree was constructed using neighbor-joining method using 71 ScAP2/ERFs, Poisson model with pairwise deletion. Bootstrap values from 1000 replicates were used to assess the robustness of the tree.

FIGURE S3 **Phylogenetic analysis of *AtDREBs* and *ScERFs*.** The gene tree was constructed using neighbor-joining method using all 63ScERFs and 57 AtDREBs represented all the subgroups, Poisson model with pairwise deletion. Bootstrap values from 1000 replicates were used to assess the robustness of the tree. The A-3 subfamily genes in Arabidopsis and *S. caninervis* were marked in red.

FIGURE S4 **Multiple sequence alignments of AP2 domains of AtERFs.** One hundred thirty-nine AtERF (including three Soloists) genes were aligned. Black and light gray shading indicate identical and conserved amino acid residues. The complete conserved amino acids residues were marked with asterisk. The three β-sheets regions and one α-helix region were labeled.
