# Supplementary figures and images for: Transcriptome-Wide Identification, Classification, and Characterization of AP2/ERF Family Genes in the Desert Moss Syntrichia caninervis
Source: Front Plant Sci. 2017 Feb 27;8:262. doi: 10.3389/fpls.2017.00262 (PMC5326779; doi:10.3389/fpls.2017.00262)

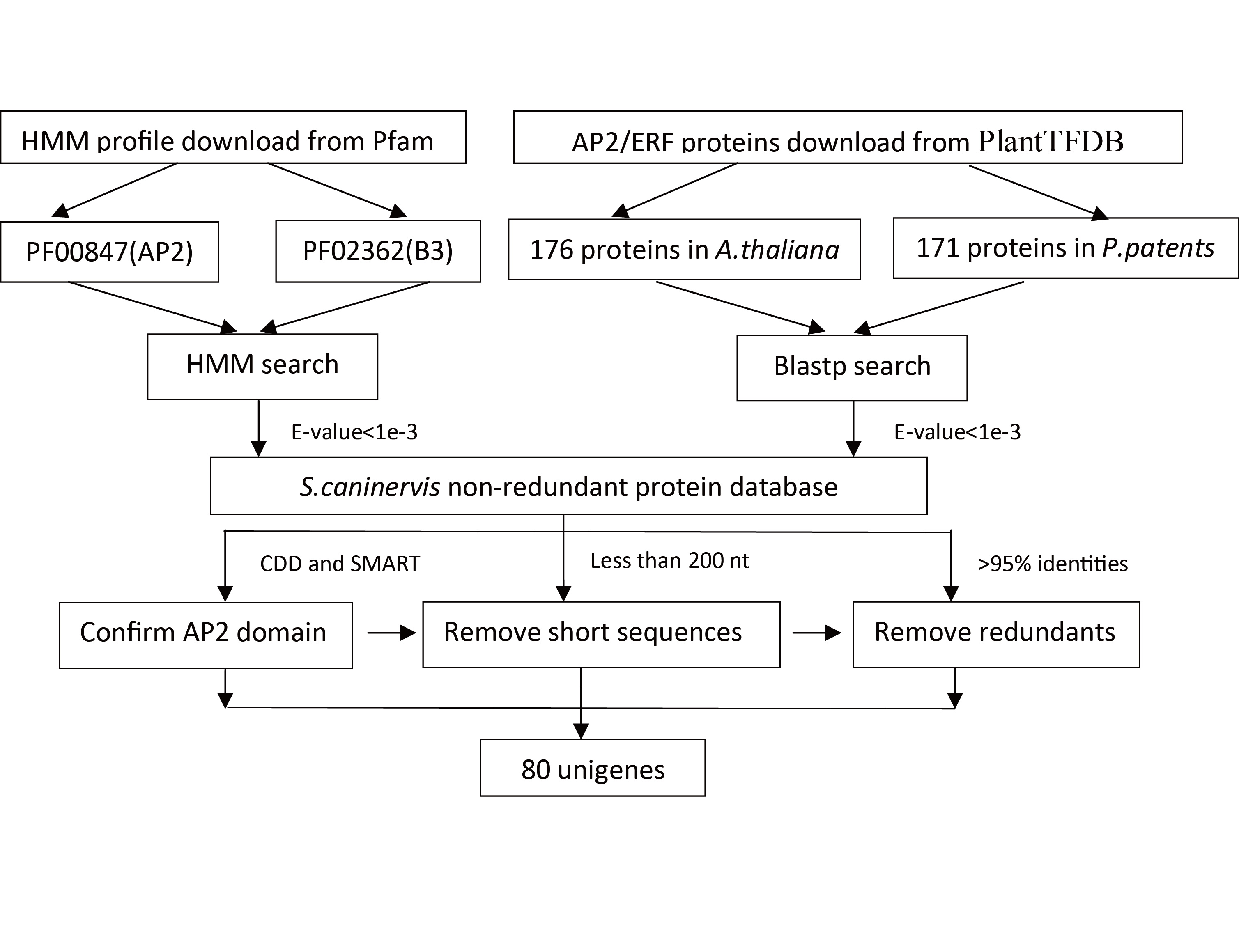

Supplement: FIGURE S1 — Flowchart of identification of AP2/ERF family genes in Syntrichia caninervis. [file Image_1.JPEG]

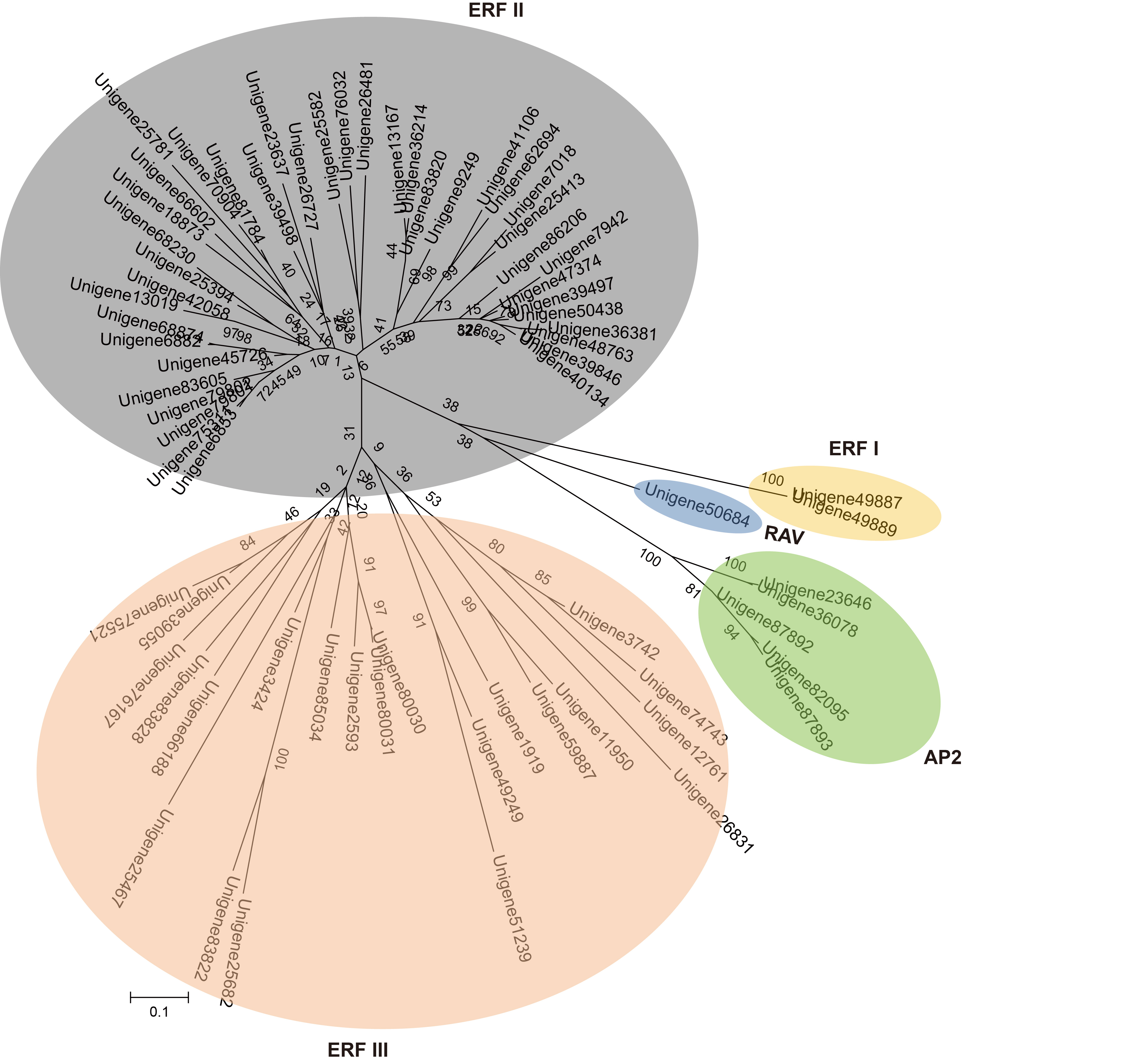

Supplement: FIGURE S2 — Phylogenetic analysis of AP2/ERF family genes in S. caninervis. The gene tree was constructed using neighbor-joining method using 71 ScAP2/ERFs, Poisson model with pairwise deletion. Bootstrap values from 1000 replicates were used to assess the robustness of the tree. [file Image_2.JPEG]

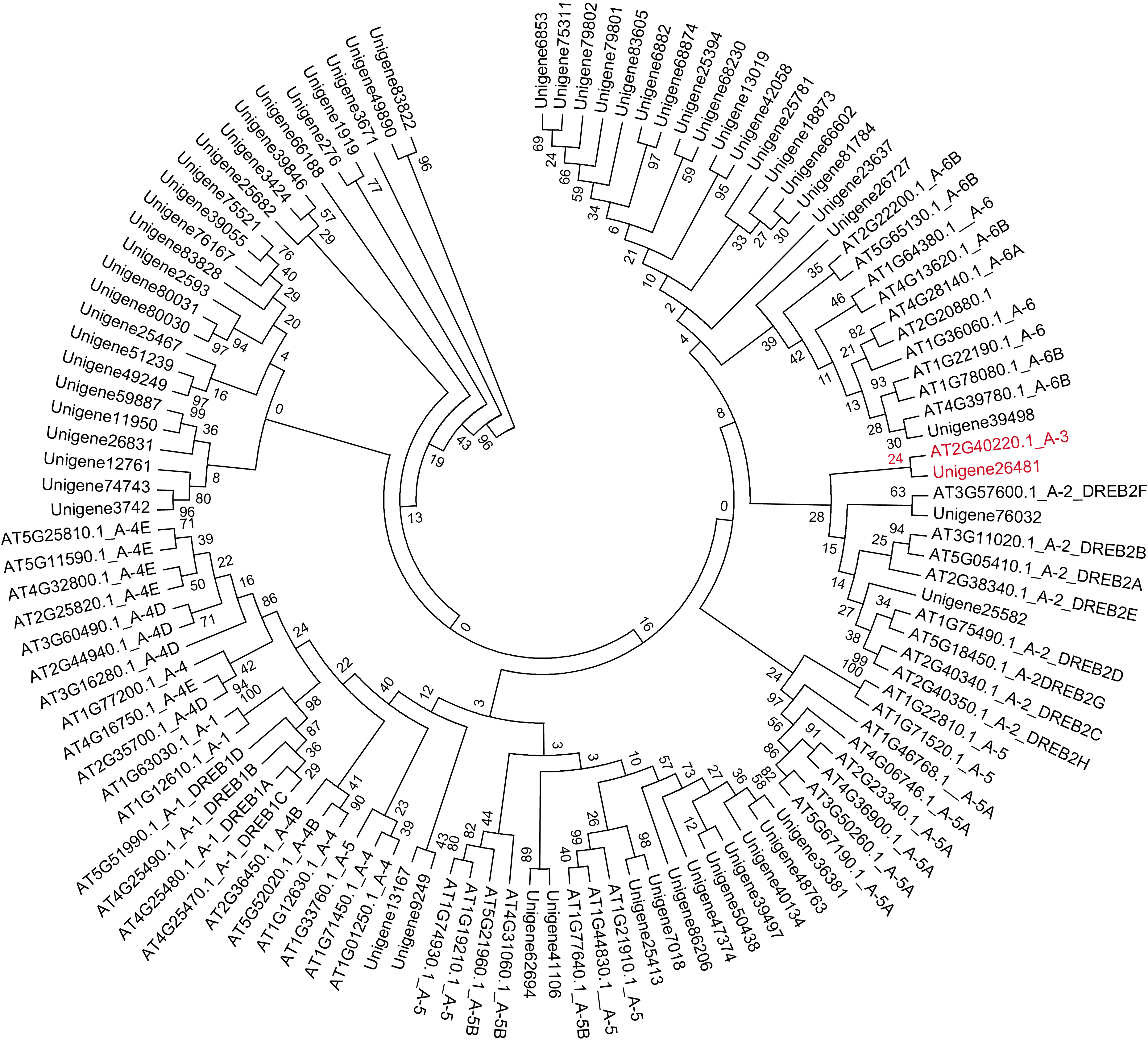

Supplement: FIGURE S3 — Phylogenetic analysis of AtDREBs and ScERFs. The gene tree was constructed using neighbor-joining method using all 63ScERFs and 57 AtDREBs represented all the subgroups, Poisson model with pairwise deletion. Bootstrap values from 1000 replicates were used to assess the robustness of the tree. The A-3 subfamily genes in Arabidopsis and S. caninervis were marked in red. [file Image_3.JPEG]

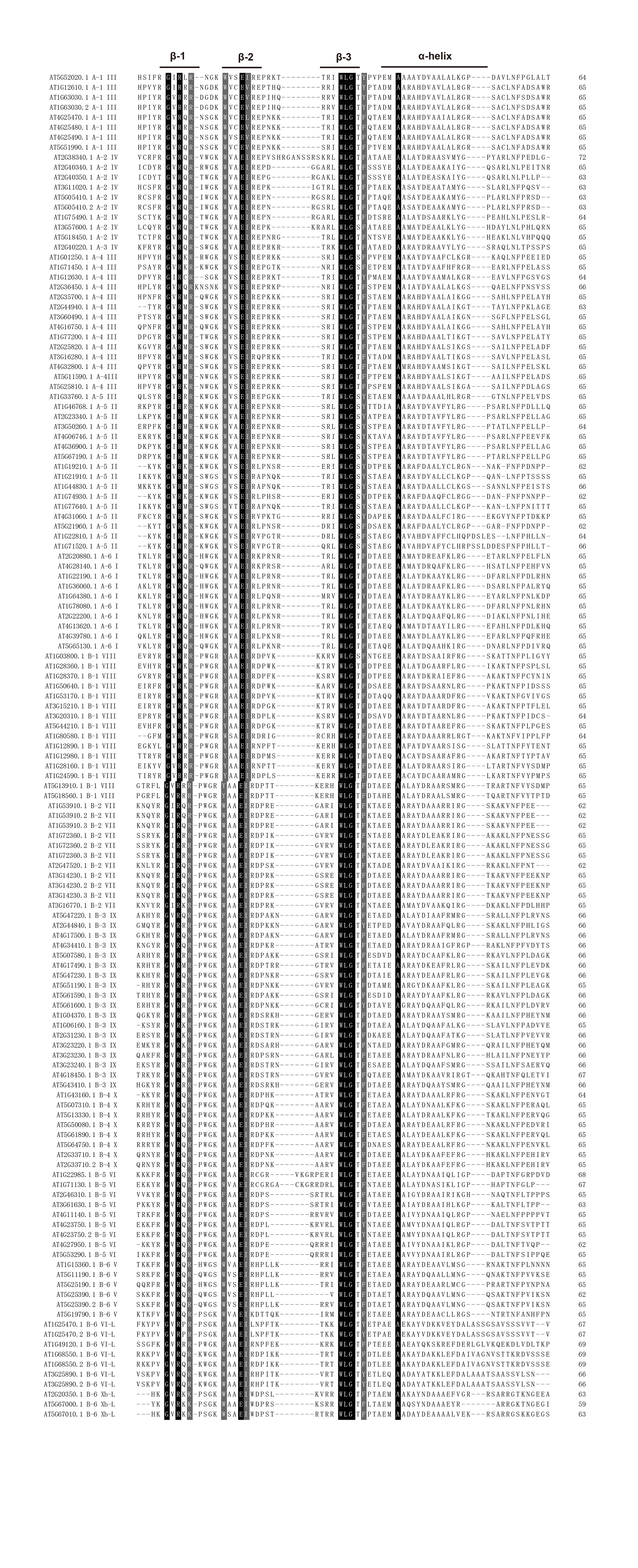

Supplement: FIGURE S4 — Multiple sequence alignments of AP2 domains of AtERFs. One hundred thirty-nine AtERF (including three Soloists) genes were aligned. Black and light gray shading indicate identical and conserved amino acid residues. The complete conserved amino acids residues were marked with asterisk. The three β-sheets regions and one α-helix region were labeled. [file Image_4.JPEG]
